# Comparing quality of life after robot assisted versus open radical cystectomy: A systematic review

**DOI:** 10.1007/s11701-025-02902-4

**Published:** 2025-10-27

**Authors:** Daniel Andrew Hawkins, Mauro Camacho, Gauri Godbole, Chiara Dell’Oro, Kingsley Ewool, Md Rezaul Karim, Bijendra Patel

**Affiliations:** 1 Queen Mary University of London, London, UK; 2 City St George’s University of London, London, UK; 3 Imperial College School of Medicine, London, UK; 4 Barts Health NHS, London, UK

**Keywords:** Bladder cancer, Robot-assisted radical cystectomy, Open radical cystectomy, Health-related quality of life (HRQoL), Patient-reported outcome measures (PROMs), Functional outcomes

## Abstract

**Background:**

Bladder cancer is a common malignancy in both sexes. Robot-assisted approaches are increasingly used in its surgical management. Radical cystectomy can significantly affect health-related quality of life (HRQoL), typically assessed using patient-reported outcome measures (PROMs) that capture patients’ physical, psychological, and social well-being.

**Objectives:**

To systematically compare HRQoL and functional outcomes following robot-assisted versus open radical cystectomy (RARC vs. ORC) for bladder cancer, and to evaluate the methodologies employed, including PROMs and postoperative assessment timepoints.

**Methods:**

A systematic search of CENTRAL, Embase, PubMed, and Scopus (2000–2025) was performed for randomised and non-randomised studies comparing HRQoL outcomes after RARC and ORC. Eligible studies reporting PROM-based HRQoL were included. Data were extracted on patient characteristics, PROMs, and outcome domains, and synthesised descriptively.

**Results:**

Nine studies involving 1,575 patients met inclusion criteria. Seven HRQoL domains were identified, assessed using 18 PROMs; the EORTC QLQ-C30 was most frequently applied (*n* = 4). HRQoL was typically assessed at 3, 6, and 12 months, with only one study extending beyond 12 months. Functional assessment was limited: one study employed the Internation Index of Erectile Function (IIEF-5) for male erectile function, none assessed female sexual function, and one reported objective continence outcomes. Across studies, no statistically significant differences in HRQoL were observed between RARC and ORC. However, heterogeneity in PROMs, inconsistent outcome reporting, and high risk of bias limited comparability.

**Conclusions:**

Current evidence does not demonstrate a HRQoL advantage of RARC over ORC. Standardisation of PROMs, consistent follow-up intervals, and inclusion of functional domains are essential to strengthen comparative evidence and inform the role of RARC as a potential new standard of care.

**Supplementary Information:**

The online version contains supplementary material available at 10.1007/s11701-025-02902-4.

## Introduction

Radical cystectomy (RC) is the standard surgical treatment for muscle-invasive bladder cancer (MIBC), and is considered in patients with high-risk non-muscle-invasive bladder cancer (NMIBC) who do not respond to intravesical Bacillus Calmette-Guérin (BCG) [1]. It can be performed via an open (ORC) or robot-assisted (RARC) approach. Randomised trials show both techniques provide comparable oncological outcomes, with RARC offering reduced blood loss but longer operative times and lower rates of neobladder construction [2, 3].

Health-related quality of life (HRQoL) is a key outcome in this population, reflecting the impact of disease and treatment on physical, psychological, and social well-being. Patient-reported outcome measured (PROMs) provide a validated means of capturing HRQoL and are increasingly used in bladder cancer research [4–6]. However, available tools vary widely in content and sensitivity, and sexual and functional domains, particularly female outcomes, are often underreported [7, 8].

Because RC involves extensive pelvic dissection, postoperative urinary continence and sexual function play key roles in determining recovery and long-term quality of life (QoL). Although RARC is hypothesised to improve short-term recovery, evidence remains conflicting, and previous systematic reviews have provided only limited exploration of PROM heterogeneity and long term outcomes [9–11].

This systematic review therefore aims to compare HRQoL and functional outcomes following RARC versus ORC, with specific focus on the PROMs employed, the postoperative timepoints assessed, and the extent to which functional domains such as continence and sexual health are captured.

## Methods

This systematic review was performed in accordance with the Preferred Reporting Items for Systematic Reviews and Meta-Analyses (PRISMA) guidelines [12] and in line with protocol agreed by all authors. The study protocol was written prior to the review being conducted and uploaded to the International Prospective Register of Systematic reviews (PROSPERO, CRD420251015798). The review aims to compare and evaluate HRQoL outcomes between RARC and ORC for bladder cancer.

### Data selection

Eligibility criteria were established in line with the population, intervention, comparison, outcomes, and study design (PICOS) framework. *Population*: adult patients with bladder cancer undergoing radical cystectomy. *Intervention*: RARC. *Comparator*: ORC. *Outcomes*: (a) Overall HRQoL findings measured by any validated tool; (b) PROMs used; (c) postoperative timepoints, (d) functional outcomes, defined as impact on urinary and sexual function following RARC/ORC. Domains of QoL were formed after the literature search to group findings. Functional outcomes included both components of HRQoL PROMS and dedicated questionnaires or methods such as continence pad measures. *Study design*: randomised-controlled trials (RCTs), prospective studies, and retrospective studies. Both randomised and non-randomised were included to broaden the scope and robustness in findings. Exclusion criteria: studies that were: (a) using paediatric populations or partial cystectomy, (b) non-comparator studies, (c) not published in English, or with no feasible translation, and (d) case reports, case series, letters, editorials, reviews, conference abstracts. See Online Resource 1 and 2 for full PICOS and Inclusion/Exclusion criteria.

A comprehensive search of existing literature was independently conducted across selected electronic databases using pre-specified, database-adapted strategies. These incorporated a combination of controlled vocabulary (e.g. MeSH terms), free-text keywords, Boolean logic, and appropriate filters to maximise sensitivity and specificity. Keywords include “Bladder cancer”, “radical cystectomy”, Robot*, “Open radical cystectomy”, “Quality of life”. Example search strategies are found in the appendix. Search dates were January 1 st 2000, to March 24th 2025. Electronic databases used for this review were PubMed (including MEDLINE), Cochrane Central, EMBASE, and Scopus. Additionally, databases of unpublished trials, including ClincialTrials.gov, WHO ICTRP and EU Clinical Trials Register were searched. The reference lists of all included studies, as well as previous reviews and meta-analyses were examined for additional trials, and the PROSPERO database was searched to locate other relevant systematic reviews. Example database searches are included in Online Resource 3.

Studies were imported into Covidence for duplication removal and screening. The selected studies were independently reviewed for eligibility by two reviewers (DAH and GG). Differences in opinion between reviewers were resolved through discussion with a third author (KE). Full texts of studies that met the inclusion criteria were retrieved and reviewed in detail.

### Data extraction

Two independent reviewers (DAH, KE) extracted data from included studies using a standardised data extraction form for evidence synthesis and quality assessment. Disagreements were solved through discussion, in which two additional authors were involved (GG, MC). In eligible studies, the following information was collected: study characteristics (first author, year of publication, country of origin, study design), patient demographics (number, average age, BMI), QoL related outcomes (HRQoL findings PROMs used, domains covered, postoperative timepoints used, and functional assessment methods when applicable). Timepoints reported in weeks were rounded to the nearest month.

### Quality assessments

Risk of bias was independently assessed by two reviewers (DAH & GG) using RoB2 and ROBINS-I for randomised and non-randomised studies respectively. Risk of bias was conducted to reflect the risk in measuring QoL outcomes. To assess overall certainty in outcomes, the Grading of Recommendations, Assessment, Development and Evaluation working group approach (GRADE) was used. Risk of bias, inconsistency, indirectness, imprecision, and publication bias was assessed across the outcomes of this review. Overall certainty was reported either as high, moderate, low, or very low depending on downgrading. Disagreements in quality assessment were discussed and resolved by discussion with a third team member when necessary (KE). Risk of bias was visualised using the ROBVIS tool [13].

### Data analysis

As conducting a meta-analysis would exclude a substantial proportion of studies, a descriptive synthesis was undertaken in accordance with the European Social Research Council guidance on narrative synthesis in systematic reviews [14]. This synthesis identifies key components and corresponding domains, and further categorises data based on statistically significant findings. Results are organised by domain, retrospectively defined from the data extraction. Additionally, PROMs, postoperative assessment timepoints, and functional evaluation methodologies are summarised.

A quantitative meta-analysis was not performed due to substantial heterogeneity among included studies. Variability in PROM instruments, follow-up durations, and reporting formats limited the feasibility of statistical pooling. Although a meta-analysis could have been undertaken by restricting inclusion to a single PROM type to enhance comparability, this would have markedly reduced the number of eligible studies and limited the scope of synthesis. Instead, a comprehensive narrative analysis was conducted to capture the full range of HRQoL outcomes across different validated instruments.

Exploratory meta-analysis was attempted by aligning outcomes to standardised timepoints (3, 6, and 12 months) and considering whether domain scores could be averaged across studies. Meta-analysis with exploratory heterogeneity testing (I²) was also considered for individual subdomains.

Due to substantial heterogeneity in PROM instruments, reporting formats, follow-up timepoints, and because pooling would risk obscuring clinically important subgroup differences, a narrative descriptive synthesis was undertaken by group consensus as the most comprehensive and clinically relevant approach.

Due to the inclusion of fewer than ten studies per comparison, formal statistical assessment of publication bias (e.g., funnel plot analysis, Egger’s test) was not undertaken. Potential for publication bias was instead considered qualitatively.

### Registration of research

This systematic review has been prospectively registered with the PROSPERO international database for systematic reviews (Registration ID: CRD420251015798). This protocol contains information on the research question, search strategy, inclusion/exclusion criteria, and risk of bias analysis.

## Results

### Description of the selected studies

**Fig. 1:**
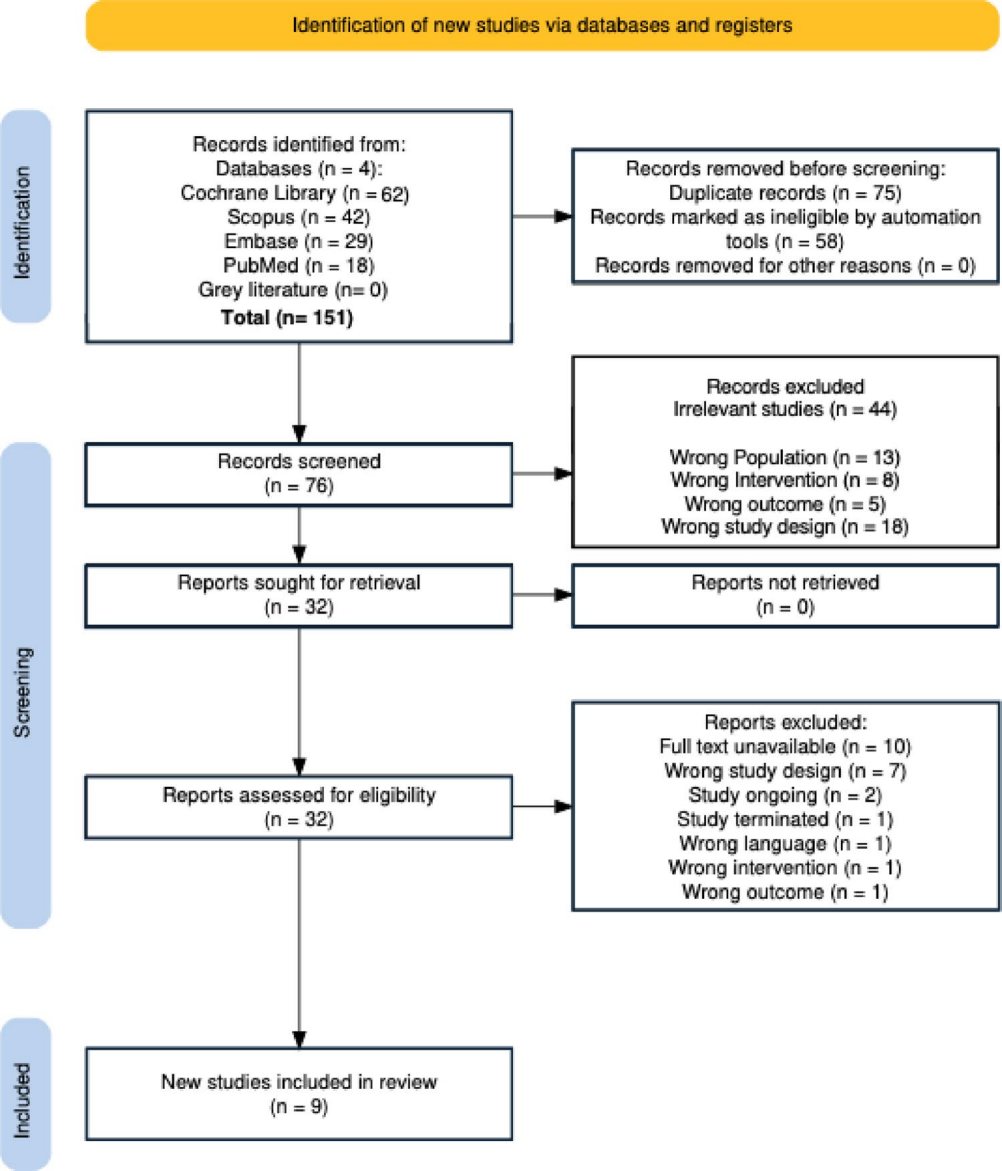
PRISMA flow diagram of the literature search and study selection process. A total of 151 records were identified through four databases. After removing duplicated and ineligible entries via automation tools, 76 records were screened. Of these, 32 full-text articles were assessed for eligibility, with 23 excluded for various reasons including study design, lack of full text, or inappropriate outcomes. Ultimately, 9 studies met inclusion criteria for this systematic review

The search process is shown in Fig. 1 as a PRISMA flow chart. A total of 151 studies were initially identified using the search criteria. Following duplicate removal, 76 studies remained. Based on the results of the screening of titles and abstracts, 44 studies were removed. Full text of 32 remaining papers retrieved and assessed for eligibility. Finally, 9 studies were selected for this systematic review [15–23], including a total of 1550 patients for analysis. All studies were published in English, as defined by the selection criteria. The aim of these studies was to compare outcomes including HRQoL between RARC (*n* = 751) and ORC (*n* = 799).

Patient characteristics can be found on Online Resource 4. Study characteristics are included in Table 1.

**Table 1:** Study characteristics of included studies

Author & year	Country	Study Design	Sample Size	Tools used	Timepoints postoperative	Themes covered	Key findings
Aboumohamed 2014	USA	Retrospective Case series (non-randomised)	182	BCI, BIS	• BCI: 1,3,6,12,18,24,30 months • BIS: 1,3,6,12,18,24,30 months	• Urogenital • Body Image & Self-perception	• [Urogenital] Sexual function favourable with ORC (time-averaged; Bonferroni correlation *p* = 0.047) • no other statistically significant differences
Bochner 2015	USA	Randomised Controlled Trial	118	EORTC QLQ-C30	• 3,6 months	• Gastrointestinal • Physical Capacity & Symptoms • Psychological Well-being • Social & Economic Impact • Global Health Status	• No statistical differences in any evaluated domain
Khan 2016	UK	Randomised Controlled Trial (3-arm: ORC, RARC, LRC)	30	FACT-BI	• No specified time	• Physical Capacity & Symptoms • Social & Economic Impact • Global Health Status	• No statistically significant relationships in QoL according to surgical arm .
Li 2016	USA	Retrospective cohort study	87	BCI, CARE	• BCI: 1,3,6,12 months • CARE2,4,6 weeks	• Urogenital function • Gastrointestinal Function • Physical Capacity & Symptoms • Psychological Well-being	• [Physical] Pain domain in CARE questionnaire favours ORC at 4 weeks (*p* = 0.05) • Differences minor and transient, statistically insignificant
Becerra 2020	USA	Multi-centre Randomised Controlled Trial (RAZOR Trial)	302	FACT-VCI, SF-8	• FACT BI CYS: 1,3,6,12 months • BCI: 1,3,6,12 months	• Physical Capacity & Symptoms • Psychological Well-being • Social & Economic Impact • Global Health Status	• [Physical, Psychological] Physical and mental summary scores of SF-8 at 6 months favour ORC • No statistically significant difference in FACT-VCI or other domains of SF-8
Wiljburg 2021	Netherlands	Prospective Comparative Effectiveness Study	348	FACT-BI-Cys, BCI	• FACT BI CYS: 1,3,6, 12 months • BCI: 1,3,6,12 months	• Urogenital function • Gastrointestinal Function • Physical Capacity & Symptoms • Psychological Well-being • Social & Economic Impact	• No statistically significant differences
Catto 2022	UK	Multicentre Randomised Controlled Trial (iROC)	317	EQ-5D-5 L, EORTC QLQ-C30, QLQ-BLM30, WHODAS 2.0	• All: 5, 12, 26 weeks	• Urogenital function • Gastrointestinal Function • Body Image & Self-Perception • Global Health Status	• [Global]: 12-week overall score in WHODAS 2.0 favours RARC (*p* = 0.01) • Differences not significant after 12 weeks
Vejlgaard 2022	Denmark	Double-blinded Randomised Feasibility Trial (BORAC)	50	EORTC QLQ-C30, EORTC QLQ-BLM30	• EORTC QLQ-C30: 3 months • EORTC QLQ-BLM30: 3 months	• Urogenital function • Gastrointestinal Function • Physical Capacity & Symptoms • Psychological Well-being • Social & Economic Impact • Body Image & Self-Perception • Global Health Status	• No significant differences domains or time
Mastroianni 2023	Italy	Single-centre RCT	116	EORTC QLQ-C30, QLQ-BLM30, IIEF-5	• All: 12 months	• Urogenital function • Gastrointestinal Function • Physical Capacity & Symptoms • Psychological Well-being • Social & Economic Impact • Body Image & Self-Perception • Global Health Status	• [Urogenital] Nighttime continence status favours ORC • [Urogenital, Body Image] 1 year body image and sexual functioning favours RARC • Other urinary symptoms comparable

#### Themes identified

Seven domains of HRQoL were retrospectively identified. Details and definitions of each domain can be found in Online Resource 5.

##### Urogenital function

Urogenital function, defined as assessment of urinary or sexual function, was assessed in 6 studies (3 non-RCTs, 3 RCTs, 1100 patients). Four studies found no statistically significant differences in urinary or sexual domains. One study favoured ORC over RARC for sexual function generally. One study favoured ORC for nighttime continence status, but favoured RARC for 1-year sexual functioning. Most studies suggest no statistically significant differences in urogenital function between RARC and ORC.

##### Gastrointestinal function

Gastrointestinal function, including bowel function, nausea/vomiting and appetite, was assessed in 6 studies (2 non-RCTs, 4 RCTs, 1036 patients). All 6 studies found no statistically significant differences in gastrointestinal function between RARC and ORC.

##### Physical capacity & symptoms

Physical Capacity & Symptoms, including physical function, activity, pain, dyspnoea, was assessed in 7 studies (2 non-RCTs, 5 RCTs, 1051 patients). Two studies favour ORC in this domain- one for superior physical capacity, the other for reduced pain. Other studies found no statistically significant difference in this domain. Overall, most studies suggest no statistically significant differences in physical capacity or symptoms between RARC and ORC.

##### Psychological well-being

Psychological well-being, including social and financial aspects, were assessed in 6 studies (2 non-RCTs, 4 RCTs, 1021 patients). One study favoured ORC for psychological well-being, specifically for cognitive function. The 5 other studies showed no significant difference. Overall, most studies show no statistically significant difference in psychological well-being between RARC and ORC.

##### Social & economic impact

Social and financial assessment was assessed in 6 studies (1 non-RCT, 5 RCTs, 965 patients). All studies found no statistically significant differences in socioeconomic impact between RARC and ORC.

##### Body image & self-perception

Body image was assessed in 4 studies (1 non-RCT, 3 RCTs, 665 patients). One study favoured RARC for body image, whilst the rest found no difference. Most studies found no statistically significant difference in body image between RARC and ORC.

##### Global health status

Six studies utilised domains that measured overall HRQoL (0 non-RCTs, 6 RCTs, 933 patients). One study favours RARC in overall HRQoL. The other 5 studies found no statistical difference. Overall, there is no statistically significant difference in global health status between RARC and ORC.

Domain coverage for each study is detailed in Table 2, with a descriptive synthesis of findings presented in Table 3.

**Table 2:**
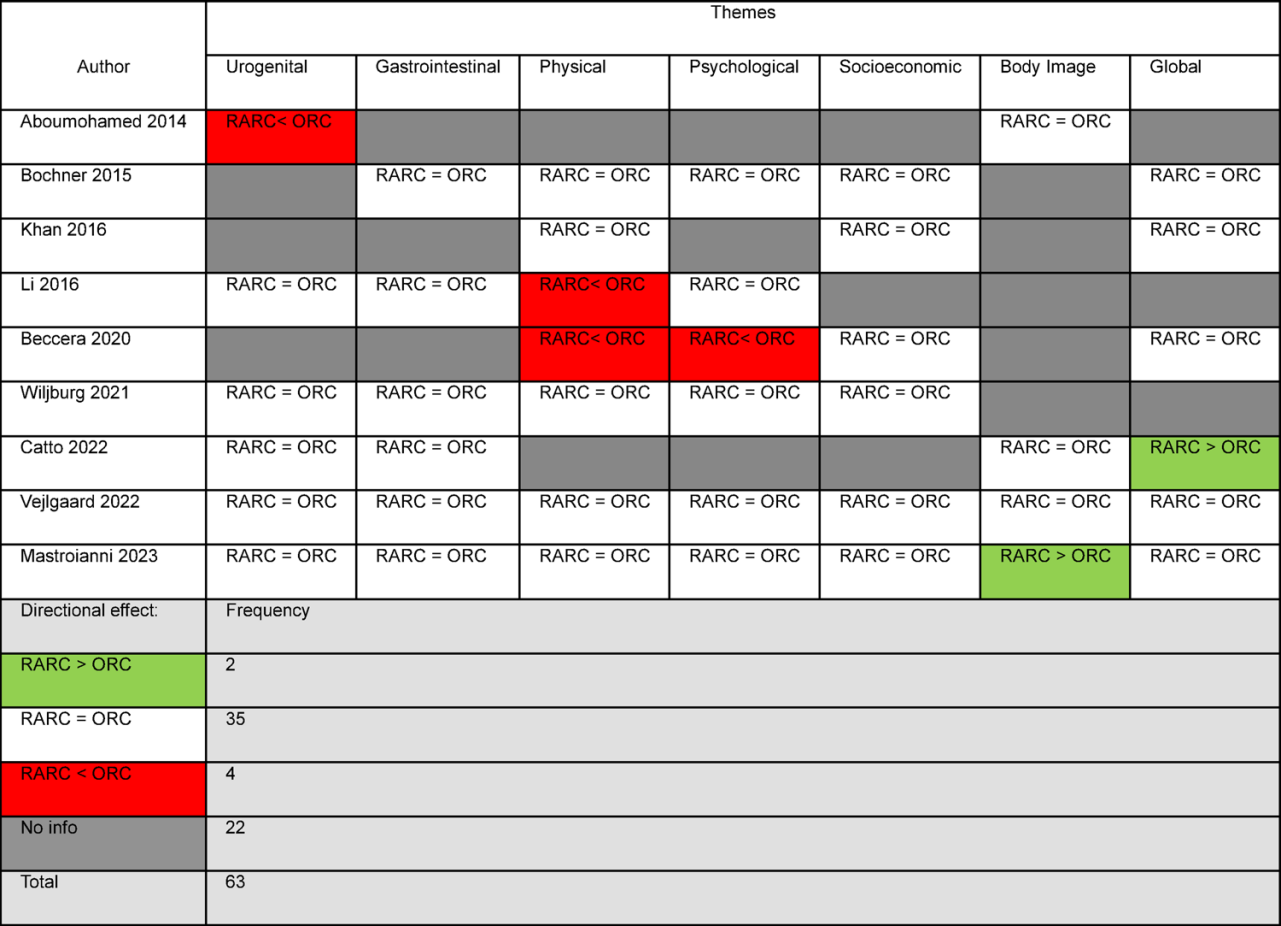
Domain Coverage of included studies. Each cell indicates the direction of effect for a given study and domain: RARC > ORC (Green), RARC = ORC (White), RARC < ORC (Red), and no information reported (Grey). Frequencies of each outcome are shown below the table

**Table 3:** Descriptive synthesis of HRQoL themes

Theme	Number of Patients (studies)	Findings	Direction of favour
Urogenital function	1100 (6)	• Majority: RARC = ORC • 1 favours ORC • 1 favoured RARC	RARC = ORC
Gastrointestinal Function	1036 (6)	• All: RARC = ORC	RARC = ORC
Physical Capacity & Symptoms	1051 (7)	• Majority: RARC = ORC • 2 favours ORC	RARC = ORC
Psychological Well-being	1021 (6)	• Majority: RARC = ORC • One favours ORC	RARC = ORC
Social & Economic Impact	965 (6)	• All: RARC = ORC	RARC = ORC
Body Image & Self-Perception	665 (4)	• Majority: RARC = ORC • 1 favours RARC	RARC = ORC
Global Health Status	933 (6)	• Majority: RARC = ORC • 1 favours RARC	RARC = ORC
Conclusion	No significant difference in HRQoL between RARC and ORC likely		

Key: RARC > ORC: RARC favoured. RARC = ORC: no difference between approaches. RARC < ORC: ORC favoured

#### PROMS used

A total of 18 questionnaire applications were identified across studies, involving 1550 total patients. The most frequently used HRQoL instruments were the EORTC QLQ-C30 (used 4 times) and the Bladder Cancer Index (BCI, used 3 times). Similarly, the EORTC QLQ-CBLM30, a bladder cancer-specific modules, was used in 3 studies with a much smaller population (*n* = 50), reflecting more limited adoption. Included PROMs with their frequencies are included in Table 4 below.

Several other questionnaires appeared only once, each with varying sample sizes. Notably used were the Body Image Scale (BIS, *n* = 182), WHO Disability Assessment Schedule (WHODAS 2.0, *n* = 317), and EQ-5D-5 L (*n* = 317), indicating occasional use of general or functional assessment tools. Instruments focusing on cancer or bladder specific QoL include the FACT-BI, FACT-BI-Cys, FACT VCI & SF-8, which combined captured data from over 680 participants.

**Table 4:** Frequency of tools used

PROM	Frequency	Number of responses:
EORTC QLQ-C30	4	540
BCI	3	854
EORTC QLQ-CBLM30	3	50
BIS	1	182
FACT BI	1	31
CARE	1	80
FACT VCI & SF8	1	302
FACT BI CYS	1	348
WHODAS 2.0	1	317
EQ-5D-5 L	1	317
IIEF	1	116
Total	18	3137

#### Timepoints measured

**Fig. 2:**
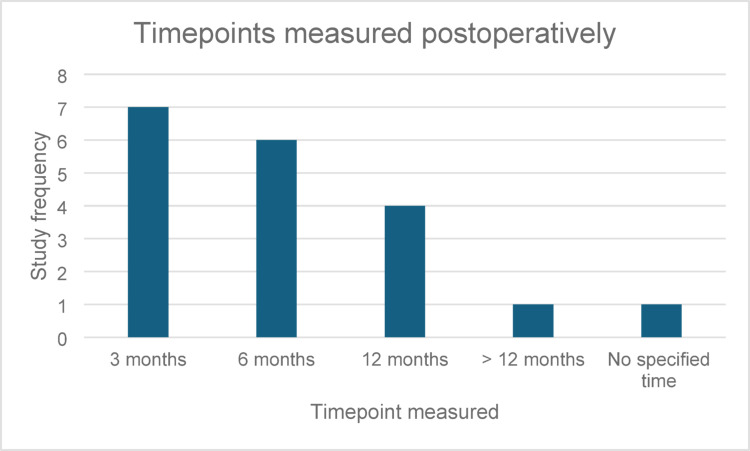
Postoperative timepoints used. The most common follow-up intervals were 3 months (*n* = 7), and 6 months (*n* = 6), with fewer studies reporting outcomes at 12 months or beyond (*n* = 1). One study did not specify a follow-up point. This variation highlights inconsistencies in longitudinal HRQoL assessment across studies

Predefined postoperative timepoints used were specified across 8 studies (3 non RCTs, 5 RCTs, 1520 patients). The most assessed timepoints were 3 months (12 questionnaires; 7 studies; 1520 patients) and 6 months (10 questionnaires; 6 studies; *n* = 1530), indicating early postoperative recovery remains a key focus for HRQoL measurement. Twelve-month HRQoL assessment was less frequent, observed in 4 studies using 8 questionnaires, suggesting a relative drop off in long term follow-up. Very few studies extended follow up beyond 12 months, with only 1 study (Aboumohamed et al., *n* = 182). One study (31 patients) did not specify timepoint. The trend reflects a predominance of short to medium term outcome measurement, with fewer studies capturing long term QoL outcomes beyond the first year. Postoperative timepoints used are shown in Table 5; Fig. 2 below.

**Table 5:** Postoperative timepoints used

Time	Questionnaire Frequency	Study frequency	Participants
3 months	12	7	1580
6 months	10	6	1530
12 months	8	4	970
> 12 months	2	1	182
No specified time	1	1	31

#### Functional outcomes

Functional outcomes of continence and sexual function were reported in 6 studies (3 non-RCTs, 3 RCTs, 1,322 patients).

##### Urinary continence

Continence outcomes were reported in 5 studies (3 non-RCTs, 2 RCTs, 1,272 patients). Most relied on continence-related subdomains within general HRQoL instruments, providing only indirect assessment. Only one study employed specific continence measures such as pad count, pad weight, or time to achieve daytime/nighttime continence. Results were inconsistent: one study reported superior nighttime continence with ORC, while the others found no significant differences between approaches.

##### Sexual function

Sexual function was assessed in 5 studies (3 non-RCTs, 2 RCTs 1020 patients), though only one employed a dedicated sexual function instrument: the International Index of Erectile Function (IIEF-5). The remainder relied on HRQoL subdomains, which provide less comprehensive assessment. Findings were inconsistent: One study favoured RARC for 1-year sexual function, whereas another favoured ORC for sexual function in aggregate analyses.

##### Female sexual function

Notably, no study evaluated female sexual function outcomes, and the Female Sexual Function Index (FSFI) [24] was not used in any cohort.

#### Quality of evidence

**Fig. 3:**
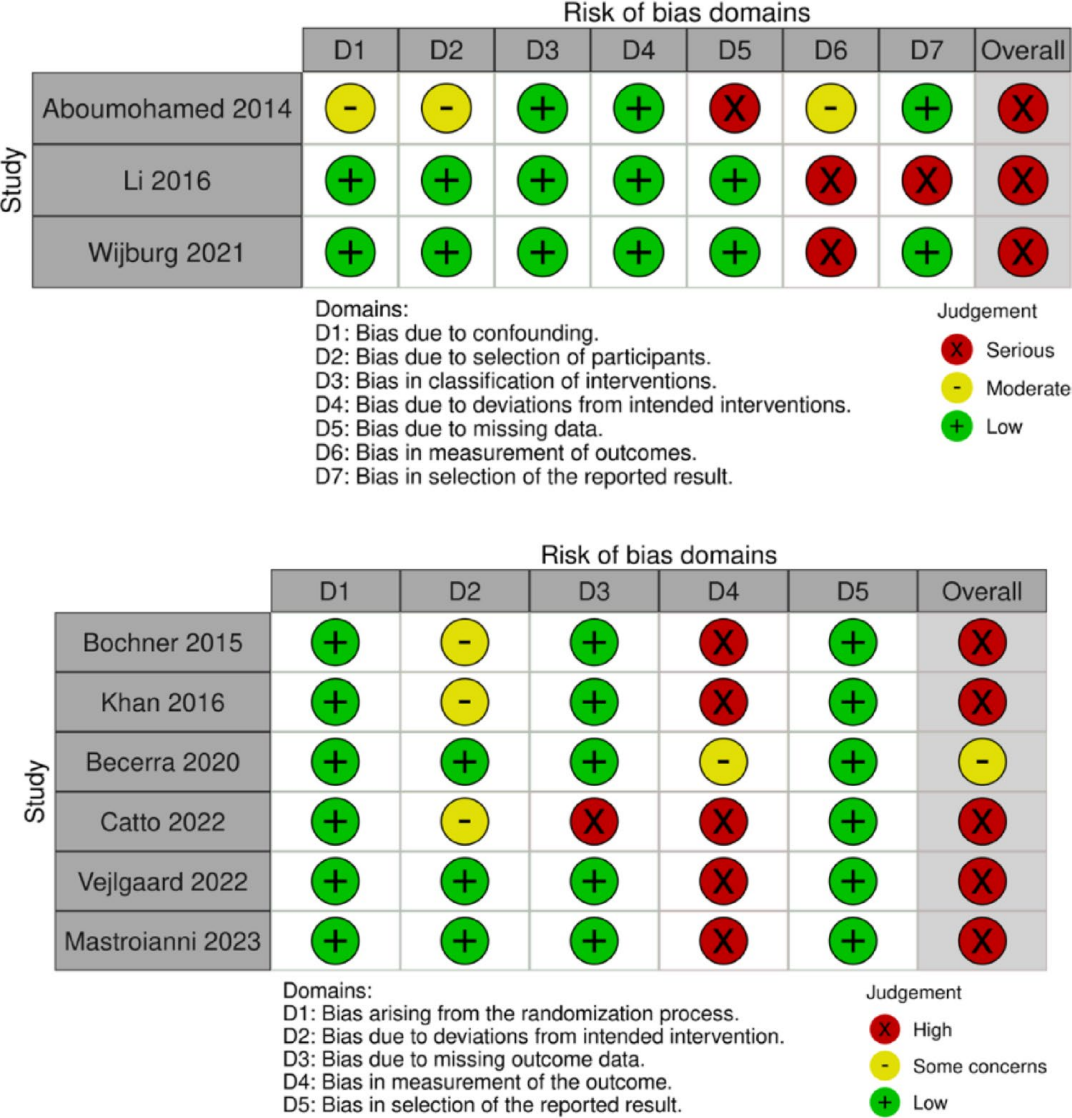
Risk of Bias assessment for non-randomised (top) and randomised (bottom) studies

Risk of bias was assessed using ROBINS-I for non-randomised studies and RoB 2 for RCTs, represented in Fig. 3 below. The most consistent source of bias was outcome measurement (D6/4), as HRQoL relies on self-reported questionnaires prone to response and performance bias. Although validated instruments aim to mitigate this, definitions of “validated” are inconsistent, adding heterogeneity. Some studies demonstrated minimal subjectivity through statistical checks, but many showed high risk from missing outcome data (D5/D3), often due to low response rates in patient-reported outcomes, where attrition can significantly influence results. Additional risks originate from confounding (e.g., ASA imbalances, surgical variables) and the difficulty of blinding in surgical trials. One feasibility RCT successfully implemented double blinding using opaque dressings changed by trial nurses, providing a rare methodological precedent for future RCTs comparing open and robotic approaches.

Certainty of evidence following the GRADE approach was rated low for Overall QoL and moderate for methodological outcomes. Risk of bias remained a consistent issue across all outcomes, with subjectivity in measurement and incomplete data affecting confidence. Overall QoL was further downgraded for indirectness due to variation in HRQoL instruments and domains assessed, resulting in partial measurement of the intended construct. The GRADE assessment can be found in Online Resource 6.

## Discussion

This systematic review synthesises the evidence on HRQoL and functional outcomes in studies comparing RARC and ORC. Overall, most findings showed no statistically significant differences across QoL domains, with isolated results favouring each approach outweighed by the consensus. The certainty of evidence was graded as low for the overall QoL findings, and moderate for methodological objectives. These findings underscore the need for high-quality, adequately powered prospective trials to enable a more definitive assessment of functional outcomes and QoL. The review also highlights substantial heterogeneity in assessment methods.

### HRQoL findings and themes identified

Across the included studies, most reported no significant differences in overall QoL between RARC and ORC. While isolated findings favoured RARC (e.g., sexual function, body image, global health) or ORC (e.g. physical capacity, psychological well-being), these were outweighed by the consensus of no meaningful difference.

We hypothesised that the minimally invasive nature of RARC would translate into higher physical recovery and body image scores; however, this was not observed across the included studies. Although RARC may reduce intraoperative blood loss, randomised trials have not consistently demonstrated further perioperative advantages, and RARC is associated with longer operative times and lower rates of neobladder diversion. Moreover, the presence of a stoma, urinary and sexual dysfunction, and long-term scarring may overshadow any cosmetic benefits of smaller incisions. In practice, the clinical relevance of cosmesis in patients undergoing radical cystectomy is limited, with long-term functional recovery, particularly continence and sexual function, having a far greater impact on QoL than scar size. This may explain why improvements in body image scores were inconsistent and generally outweighed by broader functional domains.

### Tools identified

A total of 18 different instruments were identified, with the EORTC QLQ-C30 most frequently used, followed by the Bladder Cancer Index (BCI) and the EORTC QLQ-BLM30. This wide variation in PROMs likely reflects the absence of a universally accepted HRQoL measure for cystectomy, with selection influenced by differing study aims, regional preferences, historical use, practical constraints, and inconsistent definitions of “validated” instruments. This heterogeneity underscores the lack of standardisation in HRQoL assessment following RC and presents a major barrier to meaningful comparison and synthesis of outcomes across studies.

### Postoperative timepoints

Most included studies assessed HRQoL only within the early postoperative period (1–6 months); a minority extended follow-up to 12 months, and only a single study reported outcomes beyond 1 year. This sparse long-term evidence limits inferences about recovery trajectories after 12 months. Most studies assessed HRQoL only within the first 1–6 months, likely reflecting the focus on short-term recovery, limited funding and logistics for prolonged follow-up, and the frequent designation of HRQoL as a secondary outcome, leading to a gap in long-term data.

### Functional assessment methods

Continence and sexual function were inconsistently assessed across the included studies and typically measured only through subdomains of broad HRQoL questionnaires, rather than dedicated tools. Only one study reported continence using specific measures such as pad count, pad weight, or time to achieve daytime and nighttime continence, and only one applied a validated sexual function instrument (IIEF-5). The lack of domain-specific instruments likely reflects the frequent designation of HRQoL as a secondary endpoint, the preference for shorter general questionnaires to minimise patient burden, and administrative barriers such as licensing, translation, and lack of consensus on standardised tools for cystectomy cohorts.

Importantly, female sexual function was not evaluated in any study. This omission may relate to the male predominance in bladder cancer cohorts, the absence of widely adopted female specific instruments such as the FSFI, and historical research bias toward male outcomes. Cultural sensitivities and clinician discomfort in discussing female sexual health may also contribute. Together, these gaps highlight a major weakness in the current evidence base and limit the ability of clinicians to provide comprehensive, sex-specific counselling.

Future studies should incorporate validated, procedure specific PROMs, including continence instruments such as the International Consultation on Incontinence Questionnaire (ICIQ) [25] and sex specific measures such as the IIEF-5 and FSFI, with stratification by diversion type and sex. Standardising function outcome reporting in this way would strengthen comparative research and ensure the function recovery after cystectomy is assessed with equal weight on oncological outcomes.

### Impact of bias on findings

Risk of bias was a consistent limitation across the included studies and directly affects confidence in the overall conclusions of this review. Missing outcome data was a frequent issue, with low questionnaire response rates and high attrition reducing the reliability of patient reported outcomes. As PROMs rely on voluntary completion, non-response may have disproportionately excluded patients with poorer postoperative recovery, thereby inflating HRQoL estimates.

Blinding was rarely feasible, and in most studies patients and assessors were aware of the surgical approach, introducing performance and detection bias. One randomised feasibility trial implemented double blinding with opaque dressings, demonstrating that bias can be mitigated, but this remains an exception rather than the rule.

Heterogeneity in PROMs and outcome definitions further undermines comparability. Instruments varied widely in domain coverage and sensitivity, and what was considered “validated” was inconsistent between studies. This variability not only limited the possibility of meta-analysis but also increased the risk of indirectness when synthesising results.

Taken together, these sources of bias mean that the absence of significant differences between RARC and ORC must be interpreted with caution. It is possible that true differences exist but were obscured by incomplete data, insensitive instruments, or systematic bias. Future studies should minimise attrition, adopt blinded assessment where feasible, and use agreed core outcome sets with validated instruments to reduce these risks and increase confidence in HRQoL findings.

### Narrative vs. meta-analysis

Although meta-analysis was considered, it was ultimately deemed inappropriate for this review. Exploratory approaches were attempted, including aligning outcomes to standardised timepoints (3, 6, and 12 months), averaging domain scores across studies, and assessing heterogeneity (I²) within subdomains. However, this approach would have excluded studies with non-standard or unspecified follow-up intervals and risked oversimplifying data, thereby obscuring clinically meaningful differences between domains.

Previous reviews that restricted inclusion to a single PROM instrument to enable pooling have yielded small sample sizes and limited generalisability, with few recent updates. Given the wide variability in PROM tools, outcome reporting, and follow-up durations, any pooled estimate in the present review would have been underpowered and unrepresentative of the broader evidence base.

A narrative synthesis was therefore selected as the most appropriate method, allowing inclusion of all validated PROMs and timepoints, and providing a more comprehensive and clinically relevant overview of HRQoL following RARC and ORC. From a clinical perspective, the overall trajectory of recovery and QoL across domains is of greater importance than isolated scores at individual timepoints.

### Limitations & recommendations

This review highlights several limitations in the current literature. QoL is inherently difficult to quantify as it is a multidimensional construct, and the relative importance of domains varies between patients and clinicians. Aggregating results into a single score can therefore obscure clinically meaningful differences. This challenge is amplified by the varied use of both generic and disease-specific instruments. Questionnaires differ in domain coverage, recall period, and scoring algorithms, hampering cross-study comparability and limiting the feasibility of meta-analysis. Sub-domain analyses are similarly restricted, as the same construct may be measured differently, or not at all, across tools.

To address these issues, future research should adopt a standardised HRQoL methodology, pairing a validated generic tool with domain-specific measures such as the IIEF and FSFI for sexual health and a continence-specific instrument. Consensus on core domains and tool selection could be achieved through structured, multi-stakeholder processes (e.g. Delphi consensus) and endorsed by clinical and research communities. Future studies should also be adequately powered, multicentre, and protocol-driven, with blinded outcome assessment where feasible. Standardised follow-up intervals (e.g. 3, 6, 12, and 24 months) and baseline expectations should be recorded to distinguish genuine clinical change from response shift.

While RARC offers reduced blood loss compared with ORC, broader perioperative advantages are not consistently demonstrated in randomised trials, and the impact on long-term patient-centred outcomes remains uncertain. Current HRQoL data are heterogeneous, underpowered, and frequently rely on non-standardised tools, with key domains such as urinary continence, sexual function, and female-specific outcomes underreported. Given that function recovery and QoL are central to patient decision-making, high quality, standardised, multicentre research is needed to provide definitive evidence on whether the minimally invasive advantages of RARC translate into sustained improvements in life after surgery.

## Conclusions

This systematic review highlights a significant gap in the literature regarding QoL and functional outcomes following RARC versus ORC for bladder cancer. Available studies are few, heterogeneous, and often report these outcomes only as secondary endpoints. Acknowledging the limited strength of evidence, no consistent QoL advantage was identified for either approach. While RARC offers reduced blood loss compared with ORC, randomised evidence has not confirmed broader perioperative benefits, and it is associated with longer operative times and lower rates of neobladder diversion. High quality adequately powered prospective trials with standardised methodologies are required to comprehensively evaluate function recovery and QoL, while accounting for factors such as surgical learning curve and baseline patient characteristics.

## Supplementary Information

Below is the link to the electronic supplementary material.

Supplementary Material 1

Supplementary Material 2

Supplementary Material 3

Supplementary Material 4

Supplementary Material 5

Supplementary Material 6

## Data Availability

No datasets were generated or analysed during the current study.
