# Supplementary material for: Comparing quality of life after robot assisted versus open radical cystectomy: A systematic review
Source: J Robot Surg. 2025 Oct 27;19(1):712. doi: 10.1007/s11701-025-02902-4 (PMC12554817; doi:10.1007/s11701-025-02902-4)
Supplement: Supplementary file 1 — Supplementary Material 1 [file 11701_2025_2902_MOESM1_ESM.docx]

Online Resource 1. PICOS Framework

**Population (P):** Adult patients with bladder cancer undergoing radical cystectomy (RC)

**Intervention (I):** Robotic-assisted radical cystectomy (RARC)

**Comparator (C):** Open radical cystectomy (ORC)

**Outcomes (O):**

1. **Overall QoL findings, descriptively synthesised by themes, retrospectively identifying domains after literature search**
2. **PROMs used**
3. **Postoperative timepoints used**
4. **Methods of functional assessment**
