# Supplementary material for: Comparing quality of life after robot assisted versus open radical cystectomy: A systematic review
Source: J Robot Surg. 2025 Oct 27;19(1):712. doi: 10.1007/s11701-025-02902-4 (PMC12554817; doi:10.1007/s11701-025-02902-4)
Supplement: Supplementary file 2 — Supplementary Material 2 [file 11701_2025_2902_MOESM2_ESM.docx]

Online Resource 2. Inclusion/Exclusion Criteria

| **PICOS Element** | **Inclusion** | **Exclusion** |
| --- | --- | --- |
| Population | - Adult patients (≥18 years) - Patients undergoing RARC or ORC for bladder cancer. | - Paediatric populations (<18 years) - non-bladder cancer cases - Non-human studies |
| Intervention | - Studies involving robot-assisted radical cystectomy (RARC). | - partial cystectomy - non-surgical interventions - other robotic surgeries (e.g. prostatectomy) |
| Comparison | - Studies comparing RARC to open radical cystectomy (ORC). | - Studies without a direct comparison between RARC and ORC. |
| Outcome 1: HRQoL Themes (Qualitative) | - Studies utilising HRQoL PROMs   Or using PROMS measuring Urinary or sexual functions   - Baseline QoL measurement and ≥3 month postoperatively - Studies including PROMs with either scores or reported trends provided | - Studies without QoL related PROMs or non-validated tools - Perioperative only outcomes (<90 days) |
| Outcome 2: 6-month score comparison (Quantitative) | - Studies included in Outcome 1 - Studies that report a 6-month score - Studies that provide tool used, domain and maximum score | - Studies with no 6-month score available (e.g. only describe differences in relation to comparator) - Scores that do not report the domains measured - Studies without a scale |
| Study design | - Randomized controlled trials (RCTs), cohort studies, cross sectional studies, case series - Comparator studies including ORC and RARC | - Single-arm studies - Case reports - Systematic reviews |
| Language | - Studies published in English or w/ available translation | - Foreign language without translation provided |
| Publication date | - Studies published from 2000 onward | - Studies published before 2000. |
| Surgical context | - Studies reporting standard-of-care RC with curative intent. - Intracorporeal and extracorporeal approaches to RARC | - Studies involving experimental or outdated surgical techniques not representative of current practice. |
