# Supplementary material for: Comparing quality of life after robot assisted versus open radical cystectomy: A systematic review
Source: J Robot Surg. 2025 Oct 27;19(1):712. doi: 10.1007/s11701-025-02902-4 (PMC12554817; doi:10.1007/s11701-025-02902-4)

Online Resource 3. Example database searches

| Search Name | Cochrane Central Advanced Search | |
| --- | --- | --- |
| Date run | 24/03/2025 | |
| ID | Search | Hits |
| #1 | MeSH descriptor: [Cystectomy] explode all trees | 578 |
| #2 | MeSH descriptor: [Urinary Bladder Neoplasms] explode all trees | 2427 |
| #3 | ("cystectomy"):ti,ab,kw | 2225 |
| #4 | Radical NEXT Cystectomy | 1209 |
| #5 | Radical NEXT cystectomies | 13 |
| #6 | Bladder NEXT cancer NEXT surgery | 88 |
| #7 | Urothelial NEXT carcinoma | 1139 |
| #8 | Bladder NEXT Neoplasm | 16 |
| #9 | Urothelial NEXT cancer | 538 |
| #10 | Transitional NEXT cell NEXT cancer | 26 |
| #11 | Transitional NEXT cell NEXT carcinoma | 1280 |
| #12 | #1 OR #2 OR #3 OR #4 OR #5 OR #6 OR #7 OR #8 OR #9 OR #10 OR #11 | 5191 |
| #13 | MeSH descriptor: [Robotics] explode all trees | 2117 |
| #14 | MeSH descriptor: [Minimally Invasive Surgical Procedures] explode all trees | 44097 |
| #15 | Robot* | 8950 |
| #16 | Robotic | 5728 |
| #17 | Robot | 4990 |
| #18 | Robot NEXT assisted | 3462 |
| #19 | Robotic NEXT assisted | 1420 |
| #20 | RARC | 142 |
| #21 | Minimally NEXT Invasive | 11118 |
| #22 | #13 OR #14 OR #15 OR #16 OR #17 OR #18 OR #19 OR #20 OR #21 | 59853 |
| #23 | Open NEXT Radical NEXT Cystectomy | 180 |
| #24 | Traditional NEXT Radical NEXT Cystectomy | 0 |
| #25 | Open | 194114 |
| #26 | #23 OR #24 OR #25 | 194114 |
| #27 | MeSH descriptor: [Quality of Life] explode all trees | 44954 |
| #28 | MeSH descriptor: [Urinary Incontinence] explode all trees | 3599 |
| #29 | MeSH descriptor: [Nocturnal Enuresis] explode all trees | 168 |
| #30 | MeSH descriptor: [Diurnal Enuresis] explode all trees | 17 |
| #31 | MeSH descriptor: [Absorbent Pads] explode all trees | 109 |
| #32 | MeSH descriptor: [Incontinence Pads] explode all trees | 60 |
| #33 | MeSH descriptor: [Nocturia] explode all trees | 190 |
| #34 | Quality NEXT of NEXT Life | 180163 |
| #35 | QoL | 32780 |
| #36 | HRQoL | 9068 |
| #37 | Incontinence NEXT Pads | 113 |
| #38 | Incontinence | 13401 |
| #39 | Pads | 1989 |
| #40 | Nocturia | 1615 |
| #41 | #27 OR #28 OR #29 OR #30 OR #31 OR #32 OR #33 OR #34 OR #35 OR #36 OR #37 OR #38 OR #39 OR #40 | 196245 |
| #42 | #12 AND #22 AND #26 AND #41 | 69 |
| #43 | #42 with Cochrane Library publication date Between Jan 2000 and May 2025, in Trials | |


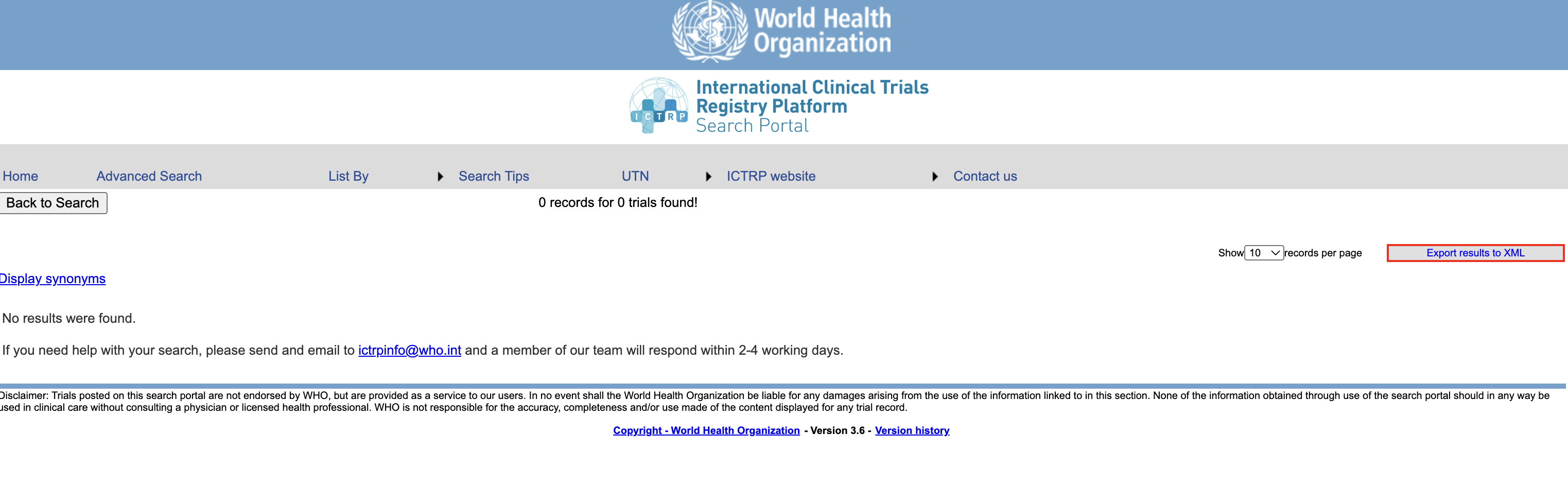


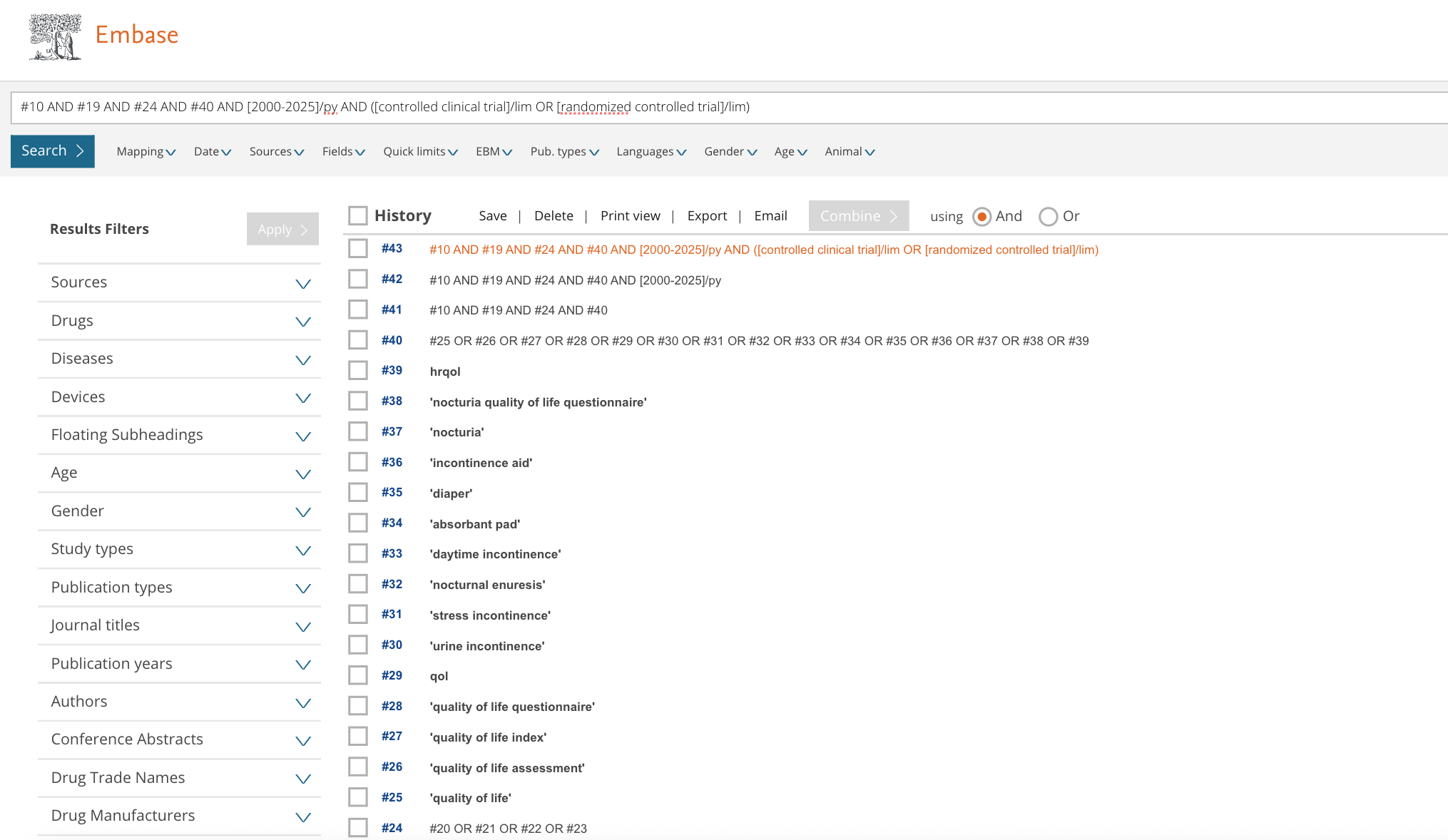


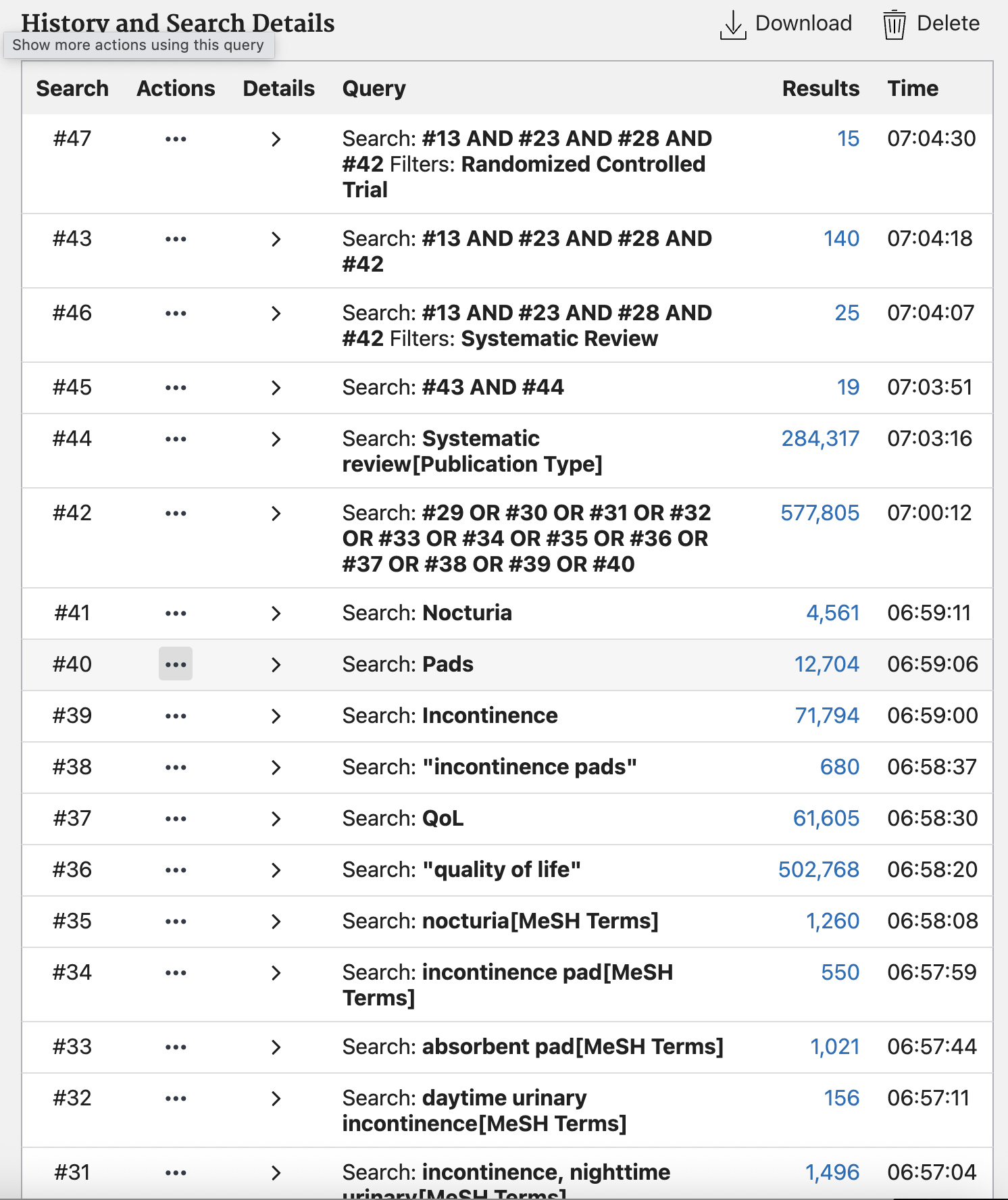


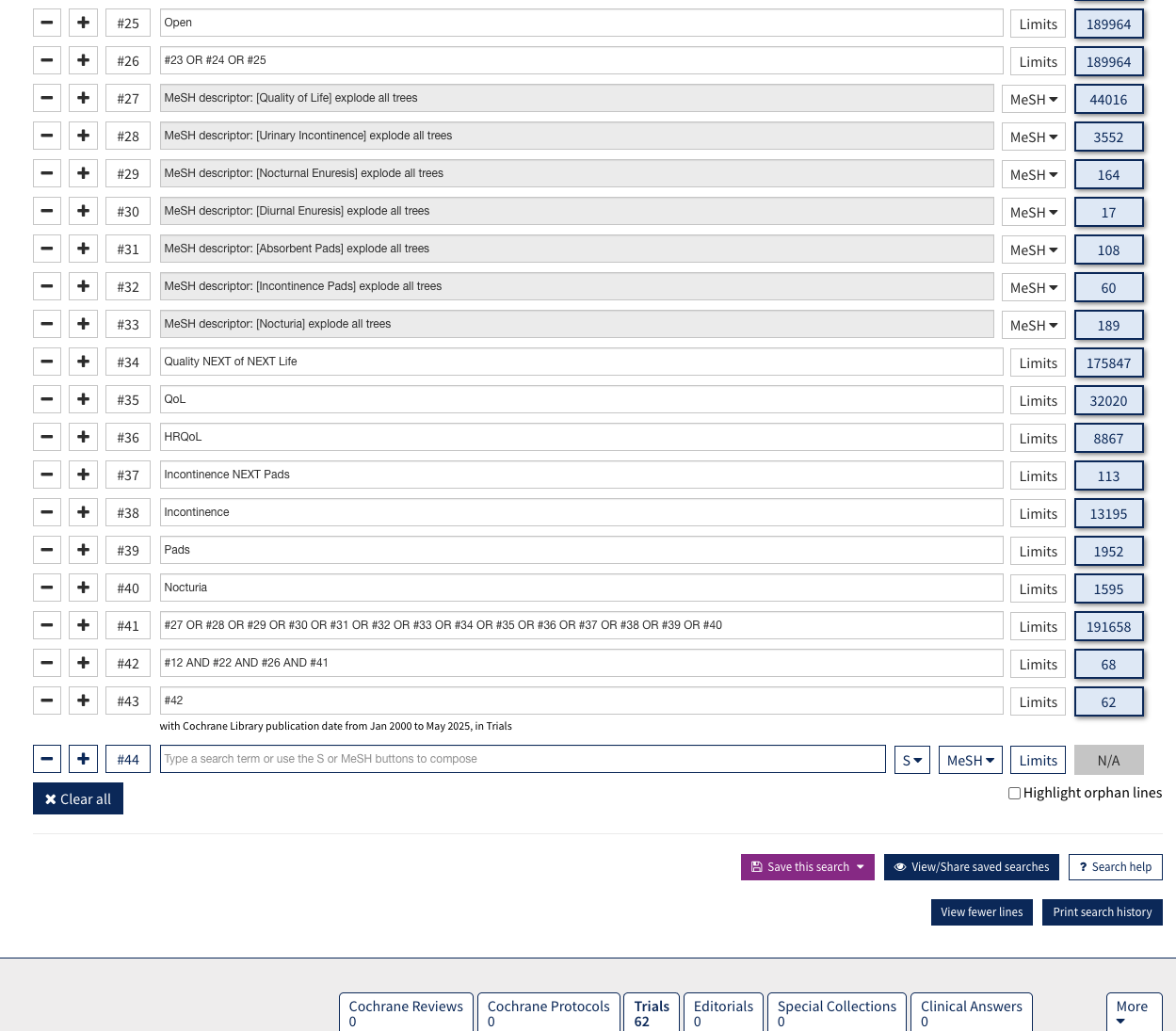

Supplement: Supplementary file 3 — Supplementary Material 3 [file 11701_2025_2902_MOESM3_ESM.docx]
