# Supplementary material for: Comparing quality of life after robot assisted versus open radical cystectomy: A systematic review
Source: J Robot Surg. 2025 Oct 27;19(1):712. doi: 10.1007/s11701-025-02902-4 (PMC12554817; doi:10.1007/s11701-025-02902-4)
Supplement: Supplementary file 4 — Supplementary Material 4 [file 11701_2025_2902_MOESM4_ESM.docx]

Online Resource 6. GRADE assessment

| Outcome | | Participants (no. of studies) | Risk of Bias | Inconsistency | Indirectness | Imprecision | Publication bias | Overall certainty |
| --- | --- | --- | --- | --- | --- | --- | --- | --- |
| 1 | Overall QoL | 1575 (9) | Serious | Not Serious | Serious | Serious | Undetected | Low ⊕⊕⊖⊖ |
|  |  |  |  |  |  |  |  |  |
| 2 | Tools used | 1575 (9) | Serious | Not Serious | Not Serious | Not Serious | Undetected | Moderate ⊕⊕⊕⊖ |
| 3 | Timepoints used | 1544(8) | Serious | Not Serious | Not Serious | Not Serious | Undetected | Moderate ⊕⊕⊕⊖ |
| 4 | Continence & Sexual function | 1322 (6) | Serious | Not serious | Not Serious | Not Serious | Undetected | Moderate ⊕⊕⊕⊖ |
|  |  |  |  |  |  |  |  |  |
