# Supplementary material for: Comparing quality of life after robot assisted versus open radical cystectomy: A systematic review
Source: J Robot Surg. 2025 Oct 27;19(1):712. doi: 10.1007/s11701-025-02902-4 (PMC12554817; doi:10.1007/s11701-025-02902-4)
Supplement: Supplementary file 5 — Supplementary Material 5 [file 11701_2025_2902_MOESM5_ESM.docx]

Online Resource 4. Patient Characteristics

| First Author, Year | Randomised/Non-Randomised | Study setting | Number of participants | | Average used | Age (years) | | Males N (%) | | BMI average | |
| --- | --- | --- | --- | --- | --- | --- | --- | --- | --- | --- | --- |
|  |  |  | RARC | ORC |  | RARC | ORC | RARC | ORC | RARC | ORC |
| Aboumohamed, 2014 | Non-Randomised | Multi-institutional (Roswell Park & Michigan) | 82 | 100 | Median (Range) | 71.5 (46-87) | 71.5 (38–91) | 64 (78%) | 70 (70%) | 27.8 | 27.9 |
| Bochner, 2015 | Randomised | Memorial Sloan Kettering  Cancer Centre | 60 | 58 | Median (IQR) | 66 (60-71) | 65 (IQR 58-69) | 51 (85%) | 42 (72%) | 27.9 | 29.0 |
| Khan, 2016 | Randomised | Guy’s Hospital, London | 15 | 15 | Mean (SD) | 68.6 (6.8) | 66.6, (8.8) | 17 (85%) | 18 (90%) | 27.5 | 27.4 |
| Li, 2016 | Non-Randomised | University of Michigan, Ann Arbor, MI, USA | 20 | 67 | Mean (SD) | 67.0 | 65.7 | 84.2% | 74.9% | N.I. | N.I. |
| Beccera, 2020 | Randomised | Multi-institutional | 150 | 152 | Mean (SD) | 68.6 (10.3) | 67.5 (9.0) | 126 (84.0%) | 128 (84.2%) | BMI (≥30, %): 34.7% | BMI (≥30, %): 23.2% |
| Wiljburg, 2021 | Non-Randomised | Dutch multicentre study | 180 | 168 | Mean (SD) | 68 (8.9) | 67 (9.5) | (78%) | (82%) | 27 | 27 |
| Catto, 2022 | Randomised | Multicentred | 161 | 156 | Median (IQR) | 68 (62-73) | 69 (62-74) | 141 (83%) | 127 (86%) | 27.2 | 27.8 |
| Vejlgaard, 2022 | Randomised | Single centre, Copenhagen University Hospital | 25 | 25 | Median (IQR) | 70 (63-74) | 67 (59-74) | 18 (72%) | 20 (80%) | 27 | 27 |
| Mastroianni, 2023 | Randomised | IRCCS “Regina Elena” National  Cancer Institute, Rome | 58 | 58 | Median (IQR) | 62 (51-66) | 63 (56-68) | 34 (74%) | 28 (67%) | 26 | 26 |
