# Supplementary material for: Comparing quality of life after robot assisted versus open radical cystectomy: A systematic review
Source: J Robot Surg. 2025 Oct 27;19(1):712. doi: 10.1007/s11701-025-02902-4 (PMC12554817; doi:10.1007/s11701-025-02902-4)
Supplement: Supplementary file 6 — Supplementary Material 6 [file 11701_2025_2902_MOESM6_ESM.docx]

Online Resource 5. QoL Domain Definition

| Theme | Constituent Domains |  |
| --- | --- | --- |
| Urogenital function | - Urinary - Sexual | Disease-specific functions most affected by cystectomy and diversion. |
| Gastrointestinal Function | - Bowel - Nausea/Vomiting - Appetite | All capture digestion-related symptoms and diet tolerance. |
| Physical Capacity & Symptoms | - Physical - Activity - Pain - Dyspnoea | Encompasses general stamina plus common post-surgical somatic symptoms. |
| Psychological Well-being | - Emotional - Cognitive | Mood, anxiety, and cognitive clarity often co-vary and are measured by similar items. |
| Social & Economic Impact | - Social - Financial | Reflect interpersonal roles, support, and cost burden. |
| Body Image & Self-Perception | - Body Image | Distinct construct frequently reported by cystectomy patients (stoma, scars, sexuality). |
| Global Health Status | - Global/Overall | Overall QoL rating that integrates multiple dimensions; keep separate so it’s not double-counted. |
